# A Review of the Efficacy and Safety of Litramine IQP-G-002AS, an *Opuntia ficus-indica* Derived Fiber for Weight Management

**DOI:** 10.1155/2014/943713

**Published:** 2014-08-28

**Authors:** Pee-Win Chong, Kai-Zhia Lau, Joerg Gruenwald, Ralf Uebelhack

**Affiliations:** ^1^InQpharm Europe Ltd., Invision House, Wilbury Way, Hitchin, Hertfordshire SG4 0TY, UK; ^2^Analyze & Realize GmbH, Waldseeweg 6, 13467 Berlin, Germany; ^3^Universitätsmedizin Charité, Campus Charité Mitte, Schumannstraße 20/21, 10117 Berlin, Germany

## Abstract

Sedentary lifestyle and caloric overconsumption are the key determinants of the escalating obesity prevalence. Reducing dietary fat absorption may help to induce a negative energy balance and thus help in managing weight problem. Apart from approved drug therapies, weight problems may also be aided with alternative and natural treatments. This paper compiled and reviewed the efficacy and safety of Litramine IQP-G-002AS, an *Opuntia ficus-indica* (OFI) derived fiber, in reducing dietary fat absorption and promoting weight loss. Evidence reviewed shows that Litramine IQP-G-002AS displays efficacy in promoting fat excretion and weight loss in four randomized, placebo-controlled clinical studies (including an unpublished pilot study). With a daily dosage of 3 g over a seven-day period, Litramine IQP-G-002AS showed an increased faecal fat excretion compared with placebo (15.8% (SD 5.8%) versus 4.6% (SD 3.1%); *P* < 0.001). In a 12-week study, significant greater weight loss (3.8 kg (SD 1.8 kg) versus 1.4 kg (SD 2.6 kg); *P* < 0.001) was observed in overweight and obese subjects treated with Litramine IQP-G-002AS as compared to placebo. No relevant gastrointestinal side effects have been reported for Litramine IQP-G-002AS at the dosages studied.

## 1. Introduction

The prevalence of overweight and obesity has increased expeditiously according to the 2012 obesity update by the Organisation for Economic Cooperation and Development (OECD) where at least one in two people is overweight or obese in more than 50% of the 34 OECD countries, while China and the United States have the largest absolute increase in the number of overweight and obese people between 1980 and 2008 followed by Mexico [1].

Obesity is associated with many noncommunicable chronic conditions, such as diabetes and cardiovascular diseases, with more than double the odds of multimorbidity compared to the nonobese [2]; a publication in 2012 reported that obesity in the United States constituted a notable 190 billion US dollar to the annual healthcare expenses [3] exceeding smoking as number one public health enemy. Therefore, effective health-care measures are necessary to reverse the obesity epidemic and to reduce soaring health care expenses.

The main cause of obesity is an energy imbalance where calories consumed exceeded calories expended [4]. In Southern Europe, the dietary fat intake is 45% and 42% of the total daily energy requirement, for male and female, respectively [5], which is approximately 8–10% higher than the recommended dietary fat intake by the European Food Safety Authority (EFSA). Such high fat dieting behavior facilitates caloric overconsumption [6]. In addition, the obesogenic environments where fast food is overabundant and hectic, sedentary lifestyles often hinder the desire of the overweight and obese individuals to maintain a healthy diet low in fat [7]. Therefore, therapeutic agents, which reduce the dietary fat absorption may be useful to help these subjects in their weight loss efforts.

Currently, there are several approaches for the reduction of dietary fat absorption; these include (1) inhibition of the digestion process of dietary fats through inhibition of pancreatic lipase, the main fat digestion enzyme, (2) binding to bile acids that slow down the digestion of fats [8], or (3) reduction of the fat absorption by restricting the physical contact between nutrients and intestinal villi [9]. Meanwhile, pharmaceutical lipase inhibitor, Orlistat, which prevents 25–30% of dietary fat from being absorbed, is the only authorized drug treatment in this category. However, approximately 15–30% of patients experience gastrointestinal side effects such as fatty or oily stool, oily spotting during the treatment, and fecal incontinence [10]. Safety concerns have been raised about a possible link between Orlistat and the risk of acute liver injury [11].

Alternative treatments for reducing dietary fat uptake have also been evaluated. One of the concepts is the use of dietary fibers [8]. Baer et al. [9] indicated that an increased consumption of dietary fiber resulted in a decrease of metabolized energy that may be attributed to the reduction of fat digestion, while a prospective cohort study in 2009 [12] further corroborated the efficacy of dietary fiber in reducing body weight and body fat. However, there seem to be major differences in fat binding properties between different fibers [13].

In this paper we compile and review evidence of effective and safe use of dietary fiber derived from* Opuntia ficus-indica*, Litramine IQP-G-002AS in weight management.

## 2. Background

*Opuntia ficus-indica* (L.) Mill. (OFI) (also known as prickly pear, cactus pear, or nopal) is a species of cacti native to arid and semiarid regions, originates from Mexico, and was propagated to other regions like the Mediterranean region and North Africa [14].

The fruit and cladode of OFI have been part of the human diet for centuries in these regions. Particularly, the cladode (or the pad of cactus) is known for its rich fiber content. The OFI cladode generally contains approximately 40–50% dry weight of dietary fibers, which consists of soluble and insoluble fiber. The soluble fiber mainly consists of mucilage, gum, pectin, and hemicellulose while the insoluble fiber consists of cellulose and a larger fraction of hemicellulose [15–17]. The fiber composition of OFI cladode is believed to be the main contributor to its reported health benefits, including improved blood glucose levels and blood lipid profiles [18–21]. However, the findings have been inconsistent [21, 22], which may be attributed to the differences in composition of the OFI cladode products. It was reported that the fiber composition of the OFI cladode changes according to age of maturity. The insoluble fiber content increases, while the soluble fiber decreases with age [23]. Apart from age of maturation, the composition and the health properties of OFI are also affected by the plant variety, cultivation condition, and post harvesting processing method [17]. Therefore, in reviewing the clinical evidence of* Opuntia ficus-indica*, it is important to identify the active composition and functional properties of the study materials applied, as they are linked to the therapeutic effects.

Recent research using a laboratory test method has shown that an OFI cladode powder (NeOpuntia, Nexira Health), standardized in its fiber content, is able to bind to dietary fat. A similar method has been used for quantifying the fat binding capacity of other fiber compounds [24]. It is postulated that, by binding to dietary fat, the fiber prevents fats from intestinal digestion process and reduces the fat absorption. The capacity of this standardized OFI powder in reducing fat absorption has also been shown in a dynamic gastrointestinal model [25]. Additionally, a pilot clinical study with 10 healthy subjects showed that subjects receiving 4.8 g a day of the standardized OFI powder experienced 27% more fat excretion, compared to placebo [26].

Based on the same fat binding mechanism, Litramine IQP-G-002AS was developed and marketed as fat binder in recent years. Litramine IQP-G-002AS is a natural fiber complex derived from OFI cladode powder, fortified with soluble fiber from* Acacia* spp. and coprocessed with cyclodextrin using a patent pending technology, thus optimizing the fat binding performance. The fat binding capacity of Litramine IQP-G-002AS is measured and standardized based on a modified* in vitro* method that simulates the gastrointestinal conditions [24].

## 3. Efficacy Review

Published and unpublished studies on Litramine IQP-G-002AS regarding its use in reducing fat absorption and weight management were identified for this systematic review. Published sources consisted of data from various databases such as PubMed, Scopus, and Google Scholar, while unpublished data was obtained from the key authors of the studies. Randomized controlled studies in both animal and human were considered for the review of efficacy.

One (1) preclinical study and four (4) clinical studies have been identified, all of which were conducted with Litramine IQP-G-002AS, between 2009 and 2013, to investigate its efficacy and safety in faecal fat excretion, weight loss, and weight loss maintenance.

### 3.1. Animal Study on Weight Gain Prevention and Fecal Fat Excretion

**Figure 1:**
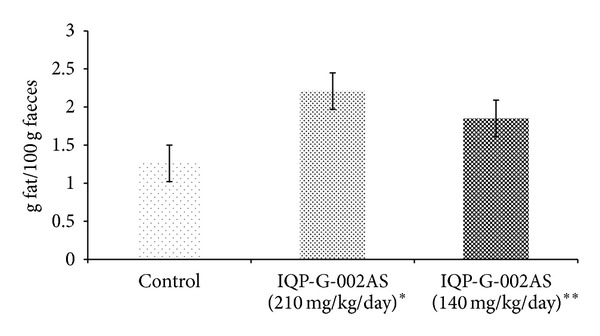
24-hour faecal fat excretion (animal study). Faecal fat excretion increased significantly in Sprague-Dawley rats fed with Litramine IQP-G-002AS in two doses, compared to control (*P* < 0.01) (*n* = 6 for control and each of the Litramine IQP-G-002AS group). Asterisk (∗) denotes statistically significant, compared to control and Litramine IQP-G-002AS (140 mg/kg/day); double-asterisk (∗∗) denotes statistically significant, compared to control. Error bars denote standard deviation.

**Figure 2:**
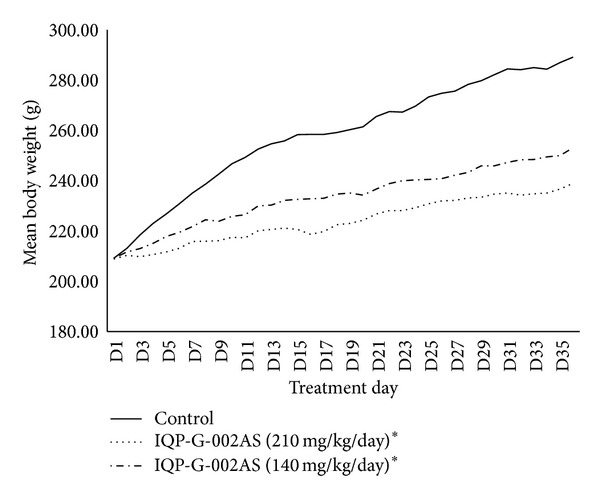
Change in body weight (animal study). Litramine IQP-G-002AS (at two doses) has shown significant efficacy in preventing body weight gain in Sprague-Dawley rats fed with high fat diet, compared to control group (*P* < 0.05) (*n* = 6 for control and each of the Litramine IQP-G-002AS group). Asterisk (∗) denotes statistically significant.

In an unpublished study with Sprague-Dawley rats, Litramine IQP-G-002AS in 2 doses (140 mg/kg/day and 210 mg/kg/day, eq. to human daily dose of 1.32 g and 2 g, resp.) significantly increased fecal fat excretion compared to the control group (Figure 1). In the same study, Litramine IQP-G-002AS also has shown significant efficacy in preventing body weight gain in rats fed with high fat diet, in comparison with the control group (Figure 2). This study provided the proof of concept of Litramine IQP-G-002AS's fat binding mechanism and provided useful information for the estimation of an effective intervention dose in weight management.

### 3.2. Pilot Faecal Fat Excretion Study in Human

**Figure 3:**
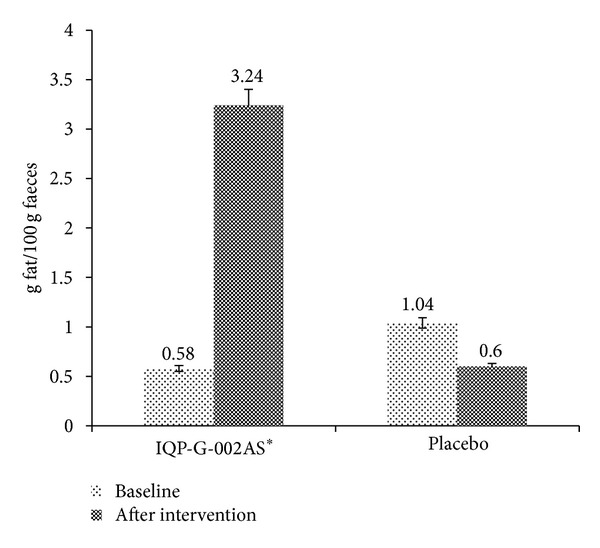
Faecal fat content before and after intake of Litramine IQP-G-002AS (2009 pilot faecal fat excretion study). Faecal fat excretion was greater in Litramine IQP-G-002AS, compared to placebo after treatment (*P* < 0.001) (*n* = 46). Asterisk (∗) denotes statistically significant, compared to placebo. Error bar denotes standard deviation.

A pilot study (unpublished) was conducted in 2009 to elucidate the dietary fat binding capacity of Litramine IQP-G-002AS. Forty-six healthy Caucasian subjects (BMI between 20 and 30 kg/m^2^) completed the two-armed, randomized, double blind, and placebo-controlled study. During the intervention phase, subjects received either 1.07 g (two tablets) of Litramine IQP-G-002AS or identical placebo for three days and were advised to adhere to a diet plan containing a daily energy intake of 2500 kcal with a fat content of 30% (80 g). Stool samples were collected on the third day and on the sixth day, for faecal fat content analysis. Compared to placebo, Litramine IQP-G-002AS has been shown to significantly increase faecal fat excretion (Figure 3). However, the study design had certain limitations as the fat content of the subject's stool was measured in aliquots only, whereas the total daily stool weight of each subject had not been measured; hence, the absolute amount of fat excreted could only be established through a theoretical calculation based on daily stool weights reported in literature.

### 3.3. Faecal Fat Excretion Clinical Study

**Figure 4:**
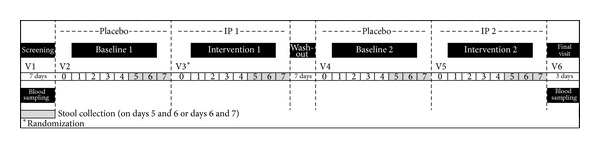
Study design summary of 2011 faecal fat excretion clinical trial.

A second faecal fat excretion trial was conducted in 2011 [27], with additional experimental conditions that addressed the corresponding study limitations, which were observed in the pilot trial. Twenty healthy Caucasian subjects (BMI between 20 and 30 kg/m^2^) completed the 45-day, double blind, randomized, and crossover fat excretion study (Figure 4). During the intervention phases, subjects received either two tablets of Litramine IQP-G-002AS (500 mg per tablet) or matching placebo tablets, thrice daily before main meals. In addition, subjects were put on a standardized diet with preprepared meals. Subjects were assigned to one of three different daily energy levels, providing 35% of the total daily energy intake by fat: 2200 kcal (with 85 g fat), 2600 kcal (with 101 g fat), and 3000 kcal (with 115 g of fat). All bowel movements within 24 hours were collected either on days 5 and 6 or on days 6 and 7 of the intervention phase. The results show that consumption of Litramine IQP-G-002AS over a period of 5-6 days increased the amount of fat excreted in the faeces. The percentage of fat excreted relative to daily fat intake (equivalent to 100%) was calculated. There was a significant (*P* < 0.001) difference between the mean percentage of dietary fat excreted in subjects on Litramine IQP-G-002AS (15.8% (SD 5.8%)) and subjects on placebo (4.6% (SD 3.1%)).

### 3.4. Weight Loss Clinical Study

A randomized, double blind, placebo-controlled trial with 30 males and 93 females overweight and obese subjects (BMI between 25 and 35 kg/m^2^) was conducted to investigate the efficacy of Litramine IQP-G-002AS in reducing body weight [28]. The subjects consumed either 3 g/day of Litramine IQP-G-002AS or placebo tablets for 12 weeks. At the end of the study, there was a statistically significant 2.4 kg greater weight loss in the Litramine IQP-G-002AS group compared to the placebo group (3.8 kg (SD 1.8 kg) versus 1.4 kg (SD 2.6 kg); *P* < 0.001). Furthermore, subjects treated with Litramine IQP-G-002AS also showed significantly greater reduction in body fat composition (0.7% (SD 1.7%) versus +0.1% (SD 2.5%); difference 0.8%; *P* < 0.031) and waist circumference (3.9 cm (SD 2.7 cm) versus 2.2 cm (SD 2.9 cm); difference 1.7 cm; *P* < 0.001) in comparison to the placebo group.

### 3.5. Follow-Up Weight Maintenance Study

Forty-nine overweight and obese adults (BMI 25–35 kg/m^2^), with documented weight loss achieved over the last three to six months prior to the study, completed the randomized, double blind, placebo-controlled weight maintenance study [29]. No dietary restriction and behavioral modification were applied; however, subjects were encouraged to maintain a nutritionally balanced diet and to continue their physical activity such as walking and cycling. In addition, subjects received either 3 g/day (1 g after each of the three meals) of Litramine IQP-G-002AS or a matching placebo. Two primary endpoints were evaluated throughout the 6-month study: (1) the difference between body weight at baseline and at final visit and (2) the maintenance of the initially lost body weight in the Litramine IQP-G-002AS group, where maintenance is defined as ≤1% weight gain.

**Figure 5:**
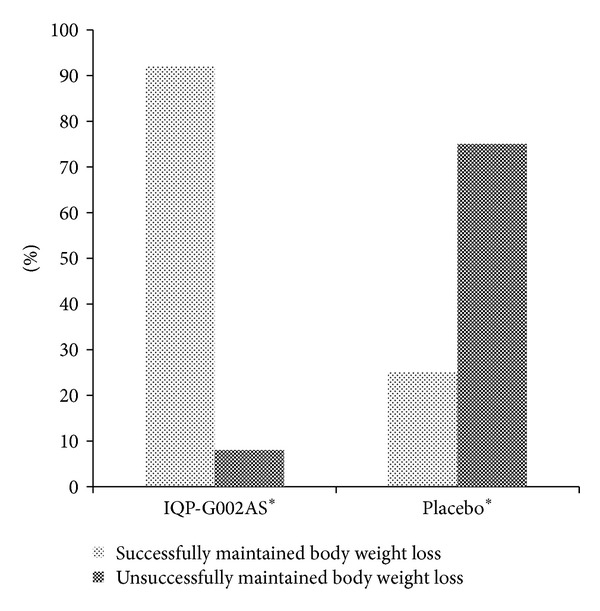
Percentage of individuals who successfully and unsuccessfully maintained body weight loss (intent-to-treat population, *n* = 49), at week 24. Asterisk (∗) denotes statistically significant (*P* < 0.001 for IQP-G002AS group; *P* = 0.012 for placebo).

Under the free-living condition, subjects in the Litramine IQP-G-002AS group lost significantly more weight than in the placebo group (−0.62 (SD 1.55) kg for IQP-G-002AS versus +1.62 (SD 1.48) kg for placebo, difference of 2.24 kg (*P* < 0.001)) at the end of week 24. In addition, significant more Litramine IQP-G-002AS subjects maintained or further reduced their body weight after initial weight loss. Conversely, more subjects in the placebo group gained weight after 24 treatment weeks (Figure 5). Moreover, improvements on waist circumference and body fat composition were observed in the Litramine IQP-G-002AS group (Table 1).

### 3.6. Fat Excretion and Weight Loss

Many products claim to bind and eliminate dietary fat manifold of their own weight in* in vitro* experiments; however the clinical efficacy remains questionable [30, 31]. The results from the above clinical studies showed that increased faecal excretion of dietary fat due to consumption of Litramine IQP-G-002AS induces weight loss, which concurs with the proposed fat binding mechanism. The weight loss effect of Litramine IQP-G-002AS may also be attributed to other functional properties of the fiber such as (1) delayed absorption of nutrients [18–20], (2) satiety promoting effects of the fiber due to the high viscosity of the fiber, which may also slow down nutrients absorption and subsequently minimize postprandial glucose spikes leading to a reduced insulin secretion [32], or (3) swelling properties of the fiber resulting in satiety signals due to stomach distension [33, 34].

## 4. Safety Review

Safety review was performed based on published and unpublished studies referring to Litramine IQP-G-002AS. Published sources consist of data from various databases such as PubMed, Scopus, and Google Scholar, while unpublished data was obtained from the manufacturers.

There were no gastrointestinal side effects reported from the clinical studies reviewed above, with the consumption of about 3 g of Litramine IQP-G-002AS a day for up to 24 weeks. There were also no clinical changes observed from blood parameters such as full blood count and clinical chemistry. Furthermore, the intake of Litramine IQP-G-002AS for 12 weeks had not resulted in any clinical relevant changes on the serum fat soluble vitamins (A, D, and E) [28] and serum mineral level (sodium, potassium, magnesium, and calcium) [28, 29]. Nevertheless, studies of more than 24 weeks are needed to examine the long-term safety of Litramine IQP-G-002AS.

## 5. Conclusion

The current review examined the efficacy and safety of Litramine IQP-G002AS, a dehydrated cladode of OFI enriched with additional soluble fiber from acacia gum and coprocessed with cyclodextrin. Published and unpublished data from randomized, placebo-controlled trials indicate positive results for faecal fat excretion, for the promotion of weight loss and for the maintenance of body weight. The safety assessment also revealed minimal concern as the studies evaluated showed that the consumption of Litramine IQP-G002AS is well tolerated. Nevertheless, further research is warranted to investigate the efficacy and safety of Litramine IQP-G002AS in reducing the risks of obesity related-comorbidities as a possible measure for health care providers and public in general to help alleviate the negative impact of the obesity epidemic.

## Figures and Tables

**Table 1:** Waist circumference and body fat mass (measured by bioimpedance analyser) changes between baseline and week 24.

Parameter	IQP-G-002AS	Placebo	*P* value
	Mean (standard deviation)	
Waist circumference (cm)	−1.7 (3.1)	+0.7 (1.5)	<0.001
Body fat mass (kg)	−1.0 (1.7)	+0.4 (1.8)	0.014

*P* value derives from the nonparametric Mann-Whitney *U* test.; positive values represent increment, while negative values represent reduction.
